# Author Correction: RNA stability controlled by m^6^A methylation contributes to X-to-autosome dosage compensation in mammals

**DOI:** 10.1038/s41594-023-01053-0

**Published:** 2023-07-06

**Authors:** Cornelia Rücklé, Nadine Körtel, M. Felicia Basilicata, Anke Busch, You Zhou, Peter Hoch-Kraft, Kerstin Tretow, Fridolin Kielisch, Marco Bertin, Mihika Pradhan, Michael Musheev, Susann Schweiger, Christof Niehrs, Oliver Rausch, Kathi Zarnack, Claudia Isabelle Keller Valsecchi, Julian König

**Affiliations:** 1 Institute of Molecular Biology (IMB), Mainz, Germany; 2 Institute of Human Genetics, University Medical Center of the Johannes Gutenberg University Mainz, Mainz, Germany; 3 Buchmann Institute for Molecular Life Sciences (BMLS) & Institute of Molecular Biosciences, Goethe University Frankfurt, Frankfurt, Germany; 4 Division of Molecular Embryology, DKFZ-ZMBH Alliance, Heidelberg, Germany; 5 STORM Therapeutics Ltd., Cambridge, UK

**Keywords:** RNA modification, RNA decay, Dosage compensation, Dosage compensation

Correction to: *Nature Structural & Molecular Biology* 10.1038/s41594-023-00997-7. Published online 18 May 2023.

In the version of this article initially published, Supplementary Table 1 was missing the “Half-life (h) DMSO” column that now appears in the updated file available in the HTML version of the article.

