# Supplementary material for: RNA stability controlled by m6A methylation contributes to X-to-autosome dosage compensation in mammals
Source: Nat Struct Mol Biol. 2023 May 18;30(8):1207–15. doi: 10.1038/s41594-023-00997-7 (PMC10442230; doi:10.1038/s41594-023-00997-7)
Supplement: Supplementary file 1 — Supplementary Methods, legends for Supplementary Tables 1–3, and Supplementary Tables 4–6. [file 41594_2023_997_MOESM1_ESM.pdf]

nature structural & molecular biology

Article https://doi.org/10.1038/s41594-023-00997-7

RNA stability controlled by m^{6}A methylation contributes to X-to-autosome dosage compensation in mammals

In the format provided by the authors and unedited

Table of content:

Supplementary Methods 2
Supplementary Tables 6
Supplementary References 11

Supplementary Methods

SLAM-seq

Statistical analysis of half-life fold changes

The influence of chromosome type on log_2-transformed fold changes in mRNA half-lives upon m^6A depletion (**Fig. 1G**) or *Mettl3* KO (**Fig. 1H**) was analysed using a categorical Gaussian linear mixed model. Distributional assumptions (normal distribution and homoscedasticity) were checked with Q-Q plots and by comparing empirical standard deviations. The factor *chromosome type* (autosome / X chromosome) was implemented as a fixed effect. To account for differences between individual chromosomes, the factor *chromosome number* (1 - 19, X) was included as a random effect. We used the R packages lme4 (v1.1.29) and lmerTest (v3.1.3). In both datasets, the fits of the random effect's variance were singular, meaning that the effect of individual chromosomes was negligible compared to the effect of chromosome type and that autosomal log_2-transformed fold changes could be pooled to form one group. Inference using Wald tests in the resulting models is equivalent to unpaired Student's *t*-tests for autosomal and X-chromosomal log_2-transformed fold changes.

Analysis of expression changes (RNA-seq)

For comparison of expression changes between groups, log_2-transformed fold changes were used. Only genes with a mean RPKM > 1 over all samples were considered. Effect sizes between groups were calculated as follows: The median log_2-transformed fold change of all autosomal genes was subtracted from the median log_2-transformed fold change of all X-chromosomal genes. This value was divided by the mean interquartile range (IQR) of both distributions, reported as the corresponding IQR of the median shift. The median shifts and IQR values for all datasets are summarised in **Table S5**.

Median X:A expression ratios were calculated using the pairwiseCI package in R using 'Median.ratio' with 10,000 bootstrap replications as described before[1]. We used categorical weighted mixed-effect Gaussian models for the analysis of RPKM levels in different cell lines (mESC male / XX / X0 and human fibroblasts / HEK293T / C643 / RPE1). We fitted the models with the R package *lme4*[2] (v1.1.29) and performed statistical inference with the R packages *lmerTest*[3] (v3.1.3) and *emmeans* (v1.8.0). A separate model was fitted for each cell line. The response variable was log-transformed mean RPKM values, filtered for mean values > 1. The factors *treatment* (DMSO and STM2457) and *chromosome type* (autosomal and X) were implemented as fixed effects. The *factor* gene ID was implemented as a random effect to account for the correlation of RPKM values belonging to the same gene. We used inverse variance weighting to account for heteroscedasticity. We used tests based on the multivariate *t*-distribution to assess for both treatments if the RPKM log-ratio between

X-chromosomal and autosomal genes was different from 1 and if the ratios were different between treatments. The P values are adjusted for multiple testing per model.

miCLIP2 to map m^{6}A sites

miCLIP2 experiment

miCLIP2 experiments in female mESC were performed as described in [4] using 1 µg of input material per replicate. For all experiments, the m^{6}A-specific polyclonal antibody from SynapticSystems (cat. 202 003) was used. 6 µg m^{6}A-specific antibody was used per 1 µg of RNA.

The miCLIP2 libraries were sequenced on an Illumina NextSeq 500 sequencing machine as 92-nt single-end reads including a 6-nt sample barcode as well as 5+4-nt unique molecular identifiers (UMIs) yielding between 32 and 46 million reads. Basic quality controls were done using FastQC (v0.11.8) (https://www.bioinformatics.babraham.ac.uk/projects/fastqc/) and reads were filtered based on sequencing qualities (Phred score) in the barcode and UMI regions using the FASTX-Toolkit (v0.0.14) (http://hannonlab.cshl.edu/fastx_toolkit/) and seqtk (v1.3) (https://github.com/lh3/seqtk/). Flexbar[5] (v3.4.0) was used to de-multiplex reads based on the sample barcode on positions 6 to 11 of the reads. Subsequently, UMI and barcode regions as well as adapter sequences were trimmed from read ends using Flexbar requiring a minimal overlap of 1 nt of read and adapter and adding UMIs to the read names. Reads shorter than 15 nt were removed from further analysis. The downstream analysis was done as described in Chapters 3.4 and 4.1 of Busch et al.[6] with an additional step to remove reads directly mapped to the chromosome ends. Those reads do not have an upstream position and, thus, no crosslink position can be extracted. Genome assembly and annotation of GENCODE[7] (release M23) were used during mapping with STAR[8] (v2.7.3a). Information on possibly occurring mutations was collected through the MD tag by running STAR with option "--outSAMattributes All".

After removing duplicates, all mutations found in reads were extracted using the Perl script parseAlignment.pl of the CLIP Tool Kit[9] (CTK, v1.1.3). The list of all found mutations was filtered for C-to-T mutations using basic Bash commands and kept in BED file format as described in [10]. Reads in this list (i.e., reads with C-to-T mutations) were removed from the de-duplicated BAM file using SAMtools[11] (v1.9) and basic Bash commands. The resulting BAM file with the truncation reads (noC2T) was transformed to a BED file using bedtools bamtobed[12] (BEDTools v2.27.1) considering only the 5′ mapping position of each read. Afterwards, the BED file was sorted and summarised to strand-specific bedGraph files, which were shifted by one base pair upstream (since this nucleotide is considered as the cross-linked nucleotide) using bedtools genomecov (BEDtools v2.27.1). All bedGraph files were transformed to bigWig track files using bedGraphToBigWig of the UCSC tool suite[13] (v365).

m^{6}A sites were predicted as described in [4]. In brief, peaks were called on noC2T reads (BAM files) using PureCLIP[14] (v1.3.1) and filtered for the presence in 3 out of 4 replicates. Then, m^{6}A sites were predicted using the machine learning model m6Aboost which we previously trained to discriminate m^{6}A sites from background in miCLIP2 data, based on data from *Mettl3* KO and control mESC. A detailed description of the method can be found in [4].

Statistical analysis of m^{6}A sites in transcripts

To analyse the m^{6}A sites in autosomes and the X chromosome, stratified by expression bins, a categorical generalized linear model for negative binomial data was fitted using the core R routine glm.nb (R version 4.1.2). The factors *chromosome type* (autosome / X chromosome) and *expression bin* (#3-8), as well as their interaction, were implemented. Based on visual assessment of the fits and on chi-squared tests for goodness of fit, the negative binomial model was selected in preference to a Poisson model. For each expression bin, Wald tests were used to test the difference between autosomes and the X chromosome. The *P* values were corrected for multiple testing (FWER-control) using the single step method implemented in the R package multcomp (v1.4.19).

To analyse the general influence of the factor *chromosome type* on m^{6}A sites, categorical generalized linear mixed models for negative binomial data were fitted using the R packages lme4 (v1.1.29) and lmerTest (v3.1.3). The factor *chromosome type* was implemented as a fixed main effect. The influences of expression bins and chromosome number were included as random effects. For the analysis of the mouse data sets, expression bins #3-8 were considered (**Figs. 3D,F and 4G**). Bins #4-9 were analysed in the HEK293T data set and bins #5-10 were analysed in the C643 data set (**Fig. 3G**). For each data set, the negative binomial models were preferable to Poisson models (visual assessment and chi-squared tests for fit of distribution). For the mouse heart data set, the likelihood ratio test and AIC comparison showed that the random effect *chromosome number* was not necessary to explain the data. The model was therefore fitted for the factors *chromosome type* and *expression bin*. The influence of the factor *chromosome type* on the m^{6}A counts was tested with Wald tests. The fitted values and 95% confidence intervals (Wald type) of the fold changes ($\log_2$) of expected m^{6}A counts in X-chromosomal over autosomal transcripts for all figures are reported in **Table S6**.

Estimation of methylation levels

Transcript annotations were taken from GENCODE (genome release M23, release 31), selecting one transcript per gene with the following hierarchy: (i) highest transcript support level, (ii) highest gene support level, and (iii) longest transcript. GGACH motifs were identified in each transcript using the R/Bioconductor package Biostrings (v2.59.2) and grep. To take into account only GGACH motifs in transcript regions with sufficient expression, we calculated the local read coverage in the miCLIP2 data. For this, the truncation reads from miCLIP2 data (noC2T reads) were converted into a single nucleotide coverage using bamCoverage (v3.5.1) from the deepTools suite[15].

The local read coverage was estimated as the median single nucleotide coverage in a 21-nt window centred on each GGACH motif. The GGACH motifs were binned by their log_2-transformed local coverage, adding a pseudo-count of 1 before log_2 transformation. Within each bin, the percentage of GGACH motifs harbouring high-confidence m^6A sites predicted by m6Aboost was calculated. Since m^6A detection partly depends on expression, this value increases steadily with increasing expression bins and then levels off at a certain methylation level. To determine this, a local linear regression curve was fitted using loess.smooth and used to identify the point at which the slope drops below 0.01 (**Extended Data Fig. 7E,F,G**). The corresponding percentage of GGACH motifs with an m^6A site was used as an estimate of the methylation level on a given chromosome. If the slope for a given chromosome did not drop below 0.01 due to coverage limitations, the percentage of methylated GGACH motifs at the transition point between bins #11 and #12 was taken to estimate the methylation level for this chromosome.

DNA-seq to determine chromosome copy numbers

DNA isolation

Cells were washed twice with ice-cold 1x PBS and collected on ice. For DNA isolation, the PureLink Genomic DNA MINI Kit (Invitrogen, 10593245) was used following the manufacturer's instructions.

DNA-seq library preparation

DNA-seq library preparation was performed by using genomic DNA, which was sheared with a Covaris E220 focused ultrasonicator. NGS library preparation was performed using half of the reaction of NEBNext Ultra II DNA Library Prep Kit for Illumina Version 6.0, 3/20 following the manufacturer's recommended protocol. Libraries were profiled on a 2100 Bioanalyzer (Agilent technologies) and quantified using the Qubit dsDNA HS Assay Kit, in a Qubit 2.0 Fluorometer (Life technologies). All samples were pooled in equimolar ratio and sequenced on an Illumina NextSeq500 sequencing device using a Mid Output flow cell as 159-nt single-end reads.

DNA-seq data processing

Basic quality controls were done for all DNA-seq samples using FastQC (v0.11.8) (https://www.bioinformatics.babraham.ac.uk/projects/fastqc/). Possibly remaining adapter sequences were trimmed using Cutadapt[16] (v2.4) prior to mapping. A minimal overlap of 3 nt between reads and adapter was required and only reads with a length of at least 20 nt after trimming (--minimum-length 20) were kept for further analysis. Reads were mapped from start to end (--end-to-end) using Bowtie2[17] (v2.3.4.3) without allowing any mismatches in a seed alignment (-N 0) of length 31 (-L 31). Additional parameters specifying the behaviour of multi-seed alignments were set as -i S,1,0.50 -D 20 -R 3. Genome assembly of GENCODE[7] release 31 (human) or release M23 (mouse) were used during mapping. Subsequently, multi-mapping or low-quality alignments were removed using SAMtools[11] (v1.9). Since sequencing of DNA samples was very shallow, detected duplicates are very likely PCR duplicates rather

than real duplicates. Thus, they were removed using Picard (v2.20.3) (https://github.com/broadinstitute/picard).

To determine copy number variations, mapped reads were counted in 100 kilobase bins for each chromosome and normalised by library size. The ratio for each bin was calculated by dividing the number of mapped reads per bin by the median of mapped reads of all bins and chromosomes. Only the canonical chromosomes 1-19 and X were considered.

Supplementary Tables

Table S1. Half-lives measured by SLAM-seq in male mESC under m^{6}A-depleted (STM2457) and control conditions. Half-lives for control and m^{6}A-depleted conditions are given for each gene with the corresponding residual standard error which indicates the goodness of the fit (see Methods). Additionally, the mean T coverage over all replicates and samples which was used for expression estimations is given for each condition.

Table S2. Summary of SLAM-seq, RNA-seq, and DNA-seq experiments conducted in this study. Table summarises the numbers of reads for all high-throughput sequencing experiments conducted in this study. For RNA-seq and DNA-seq experiments, the numbers of total sequenced reads and uniquely mapped reads are given. For SLAM-seq, the numbers of sequenced and retained read (SLAM-DUNK) are given. For miCLIP2, the numbers of uniquely mapped reads and reads after duplicate removal are given.

Table S3. Identified m^{6}A sites for miCLIP2 data on bulk female mESC. Table provides information on all m6Aboost-predicted m^{6}A sites (n = 33,371) in the miCLIP2 data performed on bulk female mESC. Coordinates are given in a bed file-compatible format, i.e., as 0-based, right-open intervals.

Provided as worksheets in Excel file Supplementary Tables.

Table S4. List of qPCR primers used to validate RNA expression upon m^{6}A depletion in male mESC. Oligonucleotides used as primers for qPCR experiments in **Extended Data Figs. 1D and 4C** are given. For each oligonucleotide, the sequence and target transcript are given together with the primer orientation (forward or reverse).

Name	Sequence 5' - 3'	Transcript	Orientation
qPCR_mNanog-for	CCTCCAGCAGATGCAAGAACTC	*Nanog*	Forward
qPCR_mNanog-rev	CTTCAACCACTGGTTTTTCTGCC	*Nanog*	Reverse
qPCR_mSox2-for	ACAGATGCAACCGATGCACC	*Sox2*	Forward
qPCR_mSox2-rev	TGGAGTTGTACTGCAGGGCG	*Sox2*	Reverse
Plp1_qPCR_for	CCAGAATGTATGGTGTTCTCCC	*Plp1*	Forward
Plp1_qPCR_rev	GGCCCATGAGTTTAAGGACG	*Plp1*	Reverse
Fmr1_qPCR_for	GGTCAAGGAATGGGTCGAGG	*Fmr1*	Forward
Fmr1_qPCR_rev	AGTTCGTCTCTGTGGTCAGAT	*Fmr1*	Reverse
Ssr4_qPCR_for	ACCACAGATCACCCCTTCTTAC	*Ssr4*	Forward
Ssr4_qPCR_rev	CCACTAACGTCGGCATAAAGAG	*Ssr4*	Reverse
Hnrnph2_qPCR_for	GGAGGGGTTCGTGGTGAAG	*Hnrnph2*	Forward
Hnrnph2_qPCR_rev	GAACACCTGATGTGCCATTTTG	*Hnrnph2*	Reverse
Itm2a_qPCR_for	TTGCCTCATACTTATGTGGTTCG	*Itm2a*	Forward
Itm2a_qPCR_rev	GCGGAAGGATTTTCGGTTGTTG	*Itm2a*	Reverse

(Continued on next page)

Table S4. List of qPCR primers used to validate RNA expression upon m^{6}A depletion in male mESC. (Continued from previous page)

Name	Sequence 5' - 3'	Transcript	Orientation
Rab11fip5_qPCR_for	CTCTGGACGAGGTCTTCCG	*Rab11fip5*	Forward
Rab11fip5_qPCR_rev	TGTTCCGTGTGAACTGGATGG	*Rab11fip5*	Reverse
Tubb3_qPCR_for	TAGACCCCAGCGGCAACTAT	*Tubb3*	Forward
Tubb3_qPCR_rev	GTTCCAGGTTCCAAGTCCACC	*Tubb3*	Reverse
Phax_qPCR_for	CGATGACGATTGCTCTCTTTGG	*Phax*	Forward
Phax_qPCR_rev	CGCATCTTGATTCTGTTCCTGG	*Phax*	Reverse
Faap100_qPCR_for	GGACGCGAGTTCGTCTATGTG	*Faap100*	Forward
Faap100_qPCR_rev	ACAGGACGTAGAGTGCCCT	*Faap100*	Reverse
Tpst2_qPCR_for	CGTGCTGTGTAACAAGGACC	*Tpst2*	Forward
Tpst2_qPCR_rev	CGTCACGCACCATTAGCAG	*Tpst2*	Reverse
qPCR_mGapdh-for	TCACCACCATGGAGAAGGC	*Gapdh*	Forward
qPCR_mGapdh-rev	CCCTTTTGGCTCCACCCT	*Gapdh*	Reverse

Table S5. Additional information for estimated effect sizes. Effect sizes for comparisons of fold changes between groups, e.g., differences in expression fold changes upon m^{6}A depletion between X-chromosomal and autosomal transcripts (**Fig. 2A**) are reported as the difference in medians of both distributions, divided by the mean interquartile range (IQR) of both distributions (see Methods). This table summarises the corresponding values for all effect sizes reported in this study, including the corresponding figure, the dataset analysed, the median shift between X-chromosomal and autosomal transcripts, the effect size, and the IQRs of distributions.

Figure	Dataset	Effect size	Median shift	IQR Chr X	IQR autosomes
Fig. 2A	mESC male RNA-seq	34%	0.11	0.31	0.35
Fig. 2D	Human primary fibroblasts RNA-seq	19%	0.08	0.4	0.47
Extended Data Fig. 4B	mESC male (3 h STM2457) RNA-seq	2%	0.0045	0.21	0.25
Extended Data Fig. 4B	mESC male (6 h STM2457) RNA-seq	27%	0.09	0.29	0.34
Extended Data Fig. 4B	mESC male (9 h STM2457) RNA-seq	22%	0.08	0.36	0.35
Extended Data Fig. 4B	mESC male (12 h STM2457) RNA-seq	21%	0.07	0.35	0.35
Extended Data Fig. 5B	Human HEK293T RNA-seq	17%	0.07	0.4	0.46
Extended Data Fig. 5B	Human C643 RNA-seq	19%	0.097	0.52	0.49
Extended Data Fig. 5B	Human RPE1 RNA-seq	18%	0.08	0.44	0.43
Extended Data Fig. 9D	mESC female X0 RNA-seq	24%	0.08	0.33	0.33
Extended Data Fig. 9D	mESC female XX RNA-seq	26%	0.08	0.31	0.32

Table S6. Additional information for statistical analyses of m^{6}A sites in transcripts. To analyse the general influence of the chromosome type on the number of m^{6}A sites in transcripts, categorical generalised linear mixed models for negative binomial data were fitted to the data (see Methods "Statistical analyses of m^{6}A sites in transcripts"). This table summarises the fitted values and 95% confidence intervals (Wald type) of the fold changes (log_2) of expected m^{6}A counts in X-chromosomal over autosomal transcripts as well as the two-tailed Wald test *P* values. The confidence intervals and *P* values in this table are not corrected for multiple testing.

**Figure**	**Fold change (log_2)**	**95% confidence interval**	***P* value**
Fig. 3D (male mESC)	-0.8178638	[-1.0904474, -0.5452803]	4.1e-09
Fig. 3F (heart)	-1.586387	[-2.065105, -1.107670]	8.34e-11
Fig. 3F (macrophages)	-1.0423472	[-1.4023045, -0.6823898]	1.38e-08
Fig. 3G (HEK293T)	-0.5777994	[-0.8826179, -0.2729808]	0.000203
Fig. 3G (C643)	-0.6506555	[-1.0391719, -0.2621391]	0.001030
Fig. 4H (bulk female mESC)	-0.6324775	[-1.0297596, -0.2351954]	0.0018

Supplementary References

1. Sangrithi, M. N. *et al.* Non-Canonical and Sexually Dimorphic X Dosage Compensation States in the Mouse and Human Germline. *Dev Cell* **40**, 289-301 e283 (2017).
2. Bates, D., Mächler, M., Bolker, B. & Walker, S. Fitting Linear Mixed-Effects Models Using lme4. *Journal of Statistical Software* **67**, 1-48 (2015).
3. Kuznetsova, A., Brockhoff, P. B. & Christensen, R. H. B. lmerTest Package: Tests in Linear Mixed Effects Models. *Journal of Statistical Software* **82**, 1-26 (2017).
4. Körtel, N. *et al.* Deep and accurate detection of m6A RNA modifications using miCLIP2 and m6Aboost machine learning. *Nucleic Acids Res* **49**, e92 (2021).
5. Roehr, J. T., Dieterich, C. & Reinert, K. Flexbar 3.0 - SIMD and multicore parallelization. *Bioinformatics* **33**, 2941-2942 (2017).
6. Busch, A., Brüggemann, M., Ebersberger, S. & Zarnack, K. iCLIP data analysis: A complete pipeline from sequencing reads to RBP binding sites. *Methods* **178**, 49-62 (2020).
7. Frankish, A. *et al.* GENCODE reference annotation for the human and mouse genomes. *Nucleic Acids Res* **47**, D766-D773 (2019).
8. Dobin, A. *et al.* STAR: ultrafast universal RNA-seq aligner. *Bioinformatics* **29**, 15-21 (2013).
9. Shah, A., Qian, Y., Weyn-Vanhentenryck, S. M. & Zhang, C. CLIP Tool Kit (CTK): a flexible and robust pipeline to analyze CLIP sequencing data. *Bioinformatics* **33**, 566-567 (2017).
10. Hawley, B. R. & Jaffrey, S. R. Transcriptome-Wide Mapping of m(6) A and m(6) Am at Single-Nucleotide Resolution Using miCLIP. *Curr Protoc Mol Biol* **126**, e88 (2019).
11. Danecek, P. *et al.* Twelve years of SAMtools and BCFtools. *Gigascience* **10**, giab008 (2021).
12. Quinlan, A. R. & Hall, I. M. BEDTools: a flexible suite of utilities for comparing genomic features. *Bioinformatics* **26**, 841-842 (2010).
13. Kent, W. J., Zweig, A. S., Barber, G., Hinrichs, A. S. & Karolchik, D. BigWig and BigBed: enabling browsing of large distributed datasets. *Bioinformatics* **26**, 2204-2207 (2010).
14. Krakau, S., Richard, H. & Marsico, A. PureCLIP: capturing target-specific protein-RNA interaction footprints from single-nucleotide CLIP-seq data. *Genome Biol* **18**, 240 (2017).
15. Ramírez, F. *et al.* deepTools2: a next generation web server for deep-sequencing data analysis. *Nucleic Acids Res* **44**, W160-165 (2016).
16. Martin, M. Cutadapt removes adapter sequences from high-throughput sequencing reads. *EMBnet.journal* **17**, 10-12 (2011).
17. Langmead, B. & Salzberg, S. L. Fast gapped-read alignment with Bowtie 2. *Nat Methods* **9**, 357-359 (2012).
