# Supplementary material for: RNA stability controlled by m6A methylation contributes to X-to-autosome dosage compensation in mammals
Source: Nat Struct Mol Biol. 2023 May 18;30(8):1207–15. doi: 10.1038/s41594-023-00997-7 (PMC10442230; doi:10.1038/s41594-023-00997-7)
Supplement: Supplementary file 2 — Reporting Summary [file 41594_2023_997_MOESM2_ESM.pdf]

Corresponding author(s): Julian König

Last updated by author(s): Feb 14, 2023

Reporting Summary

Nature Portfolio wishes to improve the reproducibility of the work that we publish. This form provides structure for consistency and transparency in reporting. For further information on Nature Portfolio policies, see our Editorial Policies and the Editorial Policy Checklist.

Statistics

For all statistical analyses, confirm that the following items are present in the figure legend, table legend, main text, or Methods section.

n/a	Confirmed	
☐	☒	The exact sample size (*n*) for each experimental group/condition, given as a discrete number and unit of measurement
☐	☒	A statement on whether measurements were taken from distinct samples or whether the same sample was measured repeatedly
☐	☒	The statistical test(s) used AND whether they are one- or two-sided<br>*Only common tests should be described solely by name; describe more complex techniques in the Methods section.*
☒	☐	A description of all covariates tested
☐	☒	A description of any assumptions or corrections, such as tests of normality and adjustment for multiple comparisons
☐	☒	A full description of the statistical parameters including central tendency (e.g. means) or other basic estimates (e.g. regression coefficient) AND variation (e.g. standard deviation) or associated estimates of uncertainty (e.g. confidence intervals)
☐	☒	For null hypothesis testing, the test statistic (e.g. *F*, *t*, *r*) with confidence intervals, effect sizes, degrees of freedom and *P* value noted<br>*Give P values as exact values whenever suitable.*
☒	☐	For Bayesian analysis, information on the choice of priors and Markov chain Monte Carlo settings
☒	☐	For hierarchical and complex designs, identification of the appropriate level for tests and full reporting of outcomes
☐	☒	Estimates of effect sizes (e.g. Cohen's *d*, Pearson's *r*), indicating how they were calculated

Our web collection on statistics for biologists contains articles on many of the points above.

Software and code

Policy information about availability of computer code

Data collection

For the following datasets, the given tools with given versions were used.
SLAM-seq processing
FastQC (v0.11.8)
SLAM-DUNK (v0.4.3)
lme4 (v1.1.29)
lmerTest (v3.1.3)

RNA Seq processing
FastQC (v0.11.8)
Cutadapt (v1.18)
STAR (v2.7.3a)
SAMtools (v1.9)
DESeq2 (v1.26.0)
Subread tool suite (v2.0.0)

miCLIP2 processing
FastQC (v0.11.8)
Flexbar (v3.4.0)
STAR (v2.7.3a)
CTK, v1.1.3)
SAMtools (v1.9)
BEDTools v2.27.1
PureCLIP (version 1.3.1)
FASTX-Toolkit (v0.0.14)

seqtk (v1.3)
bedGraphToBigWig of the UCSC tool suite (v365)

DNA seq Processing
FastQC (v0.11.8)
Cutadapt (v2.4)
Bowtie2 (v2.3.4.3)
SAMtools (v1.9)
Picard (v2.20.3)

Differential gene expression analysis
DESeq2 (v1.34.0)
lme4 (v1.1.29)
lmerTest (v3.1.3)
emmeans (v1.8.0)

GO analysis
clusterProfiler (v.4.2.2)

Comparison of m6A sites
htseq-count80 (v0.11.1)
multcomp (v1.4.19)
Biostrings (v2.59.2)
bamCoverage (v3.5.1)

Data analysis

All data analysis steps performed for this manuscript are described in detail in the manuscript. The scripts used to process the files are accessible under the GitHub repository located at: github.com/crueckle/Rueckle_et_al_2023

For manuscripts utilizing custom algorithms or software that are central to the research but not yet described in published literature, software must be made available to editors and reviewers. We strongly encourage code deposition in a community repository (e.g. GitHub). See the Nature Portfolio guidelines for submitting code & software for further information.

Data

Policy information about availability of data

All manuscripts must include a data availability statement. This statement should provide the following information, where applicable:

- Accession codes, unique identifiers, or web links for publicly available datasets
- A description of any restrictions on data availability
- For clinical datasets or third party data, please ensure that the statement adheres to our policy

All high-throughput sequencing datasets generated in this study were submitted to the Gene Expression Omnibus (GEO) under the SuperSeries accession GSE203653 (https://www.ncbi.nlm.nih.gov/geo/query/acc.cgi?&acc=GSE203653). RNA-seq data for human primary fibroblasts is available upon request.
HEK293T and C643 miCLIP data was taken from Gene Expression Omnibus with the accession number GSE163500.
m6A-seq2 data was taken from Gene Expression Omnibus with the accession number GSE178832
ChIP-seq data was taken from Gene Expression Omnibus with the accession number GSE126243

Field-specific reporting

Please select the one below that is the best fit for your research. If you are not sure, read the appropriate sections before making your selection.

☒ Life sciences ☐ Behavioural & social sciences ☐ Ecological, evolutionary & environmental sciences

For a reference copy of the document with all sections, see nature.com/documents/nr-reporting-summary-flat.pdf

Life sciences study design

All studies must disclose on these points even when the disclosure is negative.

Sample size

Samples sizes were chosen based on accepted practises in the field and stated in each figure or figure legend with at least three biological replicates. Example references from the field:
miCLIP2: Körtel N, Rücklé C, Zhou Y, et al. Deep and accurate detection of m6A RNA modifications using miCLIP2 and m6Aboost machine learning. Nucleic Acids Res. 2021;49(16):e92. doi:10.1093/nar/gkab485
RNA-seq: Yankova E, Blackaby W, Albertella M, et al. Small-molecule inhibition of METTL3 as a strategy against myeloid leukaemia. Nature. 2021;593(7860):597-601. doi:10.1038/s41586-021-03536-w
SLAM-seq: Herzog VA, Reichholf B, Neumann T, et al. Thiol-linked alkylation of RNA to assess expression dynamics. Nat Methods. 2017;14(12):1198-1204. doi:10.1038/nmeth.4435
Rothamel K, Arcos S, Kim B, et al. ELAVL1 primarily couples mRNA stability with the 3' UTRs of interferon-stimulated genes. Cell Rep. 2021;35(8):109178. doi:10.1016/j.celrep.2021.109178

Data exclusions

For RNA-seq in HEK293T cells, one replicate of DMSO condition was exluded. Gene body coverage for this sample indicated RNA degradation

Data exclusions	prior to RNA-seq library preparation. Otherwise, no data was exluded.
Replication	Each experiment was performed in at least three replicates unless stated otherwise. All replictaion attempts were successfull.
Randomization	Samples were collected to study groups by genotype and/or treatment condition. No randomization was applied.
Blinding	Blinding was not relevant due to the objective readouts (e.g. RNA-seq, SLAM-seq, qPCR) and investigators were not blinded. Investigators were not blinded. No placebo effects were expected since no patients were involved.

Reporting for specific materials, systems and methods

We require information from authors about some types of materials, experimental systems and methods used in many studies. Here, indicate whether each material, system or method listed is relevant to your study. If you are not sure if a list item applies to your research, read the appropriate section before selecting a response.

Materials & experimental systems

n/a	Involved in the study
☐	☒ Antibodies
☐	☒ Eukaryotic cell lines
☒	☐ Palaeontology and archaeology
☒	☐ Animals and other organisms
☐	☒ Human research participants
☒	☐ Clinical data
☒	☐ Dual use research of concern

Methods

n/a	Involved in the study
☒	☐ ChIP-seq
☒	☐ Flow cytometry
☒	☐ MRI-based neuroimaging

Antibodies

Antibodies used	m6A antibody, Synaptic Systems (cat. number: 202 003) RRID:AB_2279214. 6 µg of antibody was used per 1 µg of input RNA per replicate.
Validation	The used m6A antibody was obtained from a commercial vendor which ensures the quality of the antibody. In a previous publication (PMID: 34157120) the antibody was validated for used application.

Eukaryotic cell lines

Policy information about cell lines

Cell line source(s)	HEK293T were purchased from CSL (order number: Cryovial: 300192 Vital: 330192). C643 were purchased from CSL RRID:CVCL 5969. RPE1 cells were purchased from ATCC (order number: CRL-4000). Males mESC were provided by Dan Dominissini. Female mESC (TX1072) were provided by Edith Heard: TX1072
Authentication	Cells were not authenticated after the purchase.
Mycoplasma contamination	All cell lines are monitored and tested negative for Mycoplasma contamination.
Commonly misidentified lines (See ICLAC register)	No commonly misidentified cell lines were used in this study.

Human research participants

Policy information about studies involving human research participants

Population characteristics	The donor of human fibroblasts was a healthy 25 year old male proband.
Recruitment	The recruitment as control participants in a study on neurodegenerative diseases.
Ethics oversight	Ethical approval by the local ethical committee was obtained (No. 4485), and consent for research use in an anonymised way was given. The ethical approval given by the ethical committee of the University medicine at the Johannes Gutenberg University in Mainz, Germany, No. 4485

Note that full information on the approval of the study protocol must also be provided in the manuscript.
